# Exacerbated Psoriasis as a Rare Trigger of Multilocular Pyoderma Gangrenosum: A Case Report of a Rare Coincidence

**DOI:** 10.1177/1534734621990297

**Published:** 2022-02-28

**Authors:** Wiebke Sondermann, Laura Fischer, Eva Hadaschik, Joachim Dissemond

**Affiliations:** 1 Department of Dermatology, Venereology and Allergology, University Hospital Essen, University Duisburg-Essen, Essen, Germany

Dear Editor,

We report on a 71-year-old woman who presented herself in our outpatient clinic. She reported an acute exacerbation of her psoriasis vulgaris that existed for 2 years. Some of the plaques that had worsened in the context of the exacerbation had become very painful, erosive, and ulcerative on the lower legs within the last 4 weeks. Previously, psoriasis was treated with a topical combination therapy of calcipotriol and betamethasone with good results. Apart from stress, no other triggering factor explaining the current worsening of psoriasis was identified. Prior to the presentation at our outpatient clinic, oral antibiotic therapy was initiated by the general practitioner to treat the ulcerations but had not shown any lasting therapeutic effect. Her medical history includes arterial hypertension, breast cancer, malignant melanoma, and y-prosthetics operation.

**Figure 1.**
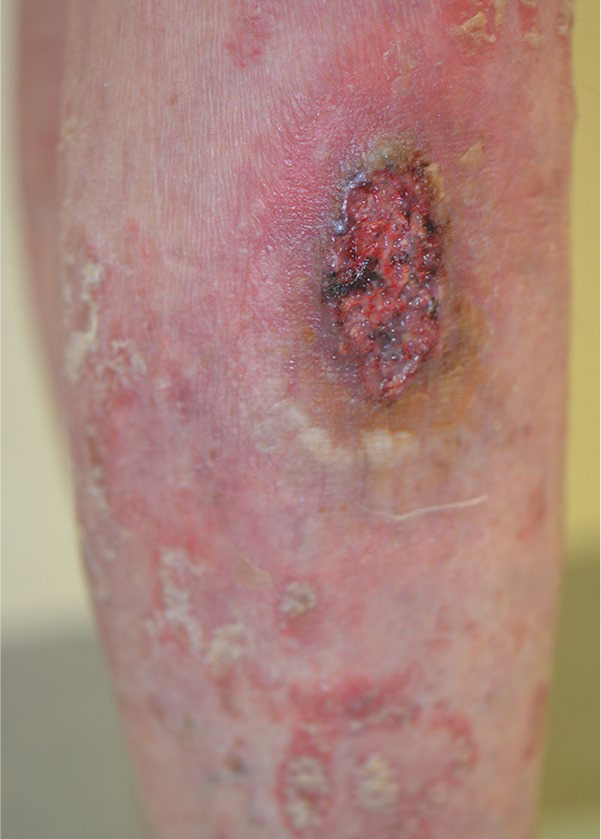
Pyoderma gangrenosum on the right lower leg.

**Figure 2.**
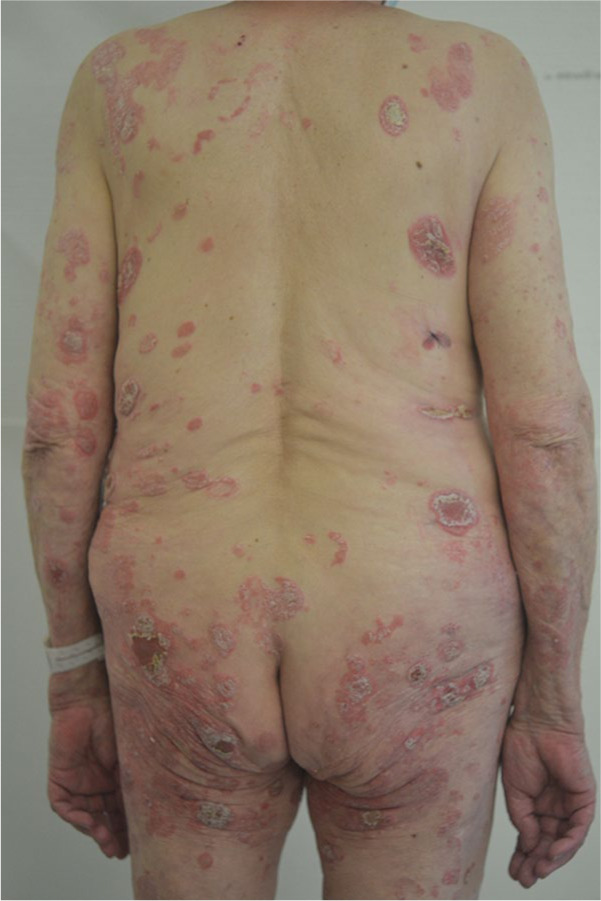
Generalized psoriatic plaques at the patient’s back accentuated in the gluteal region and on the extensor sides of the upper extremities.

**Figure 3.**
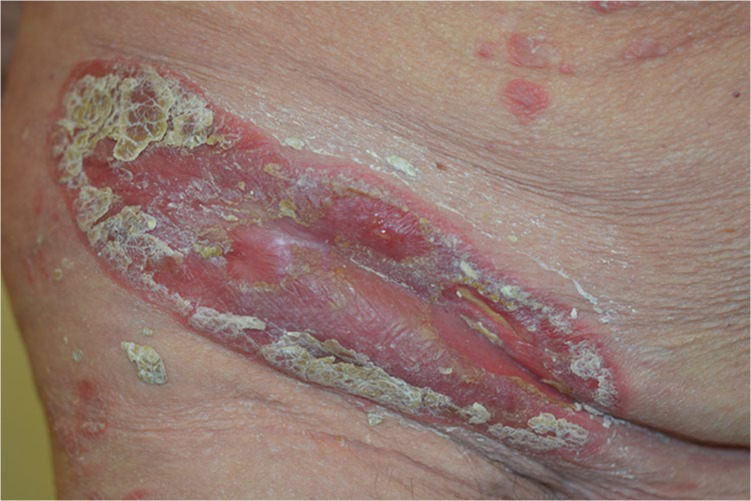
Weeping erosive plaque in the right groin clinically indicative of an initial pyoderma gangrenosum on a psoriasis plaque.

Clinical examination revealed 2 ulcers with red-violaceous borders on the left lower leg surrounded by an erythema and another isomorphic ulceration on her right lower leg (5 × 3 cm; Figure 1). Hyperkeratotic erythematosquamous plaques were present across the whole integument (Figure 2). The examination of the body folds revealed a weeping, very painful erosive plaque (20 × 10 cm) in the right groin, which was clinically consistent with an initial pyoderma gangrenosum (PG) on a psoriasis plaque (Figure 3). However, a bacterially superinfected psoriasis plaque could not be ruled out with certainty. In a swab taken from an ulceration no pathogenic bacteria were detected.

**Figure 4.**
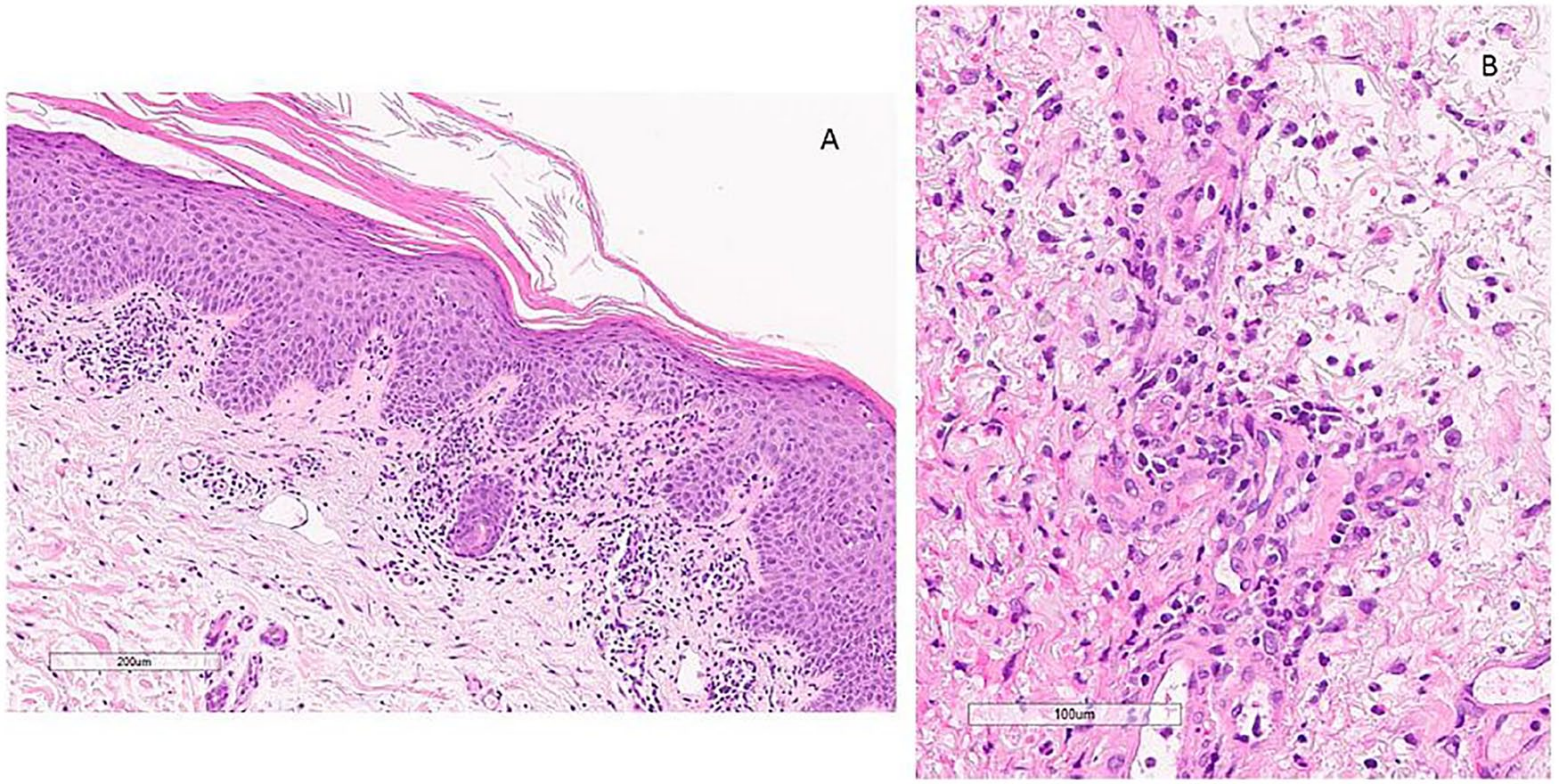
Histopathological images. (A) Biopsy findings revealed a psoriasiform acanthosis with hypogranulosis, compact parakeratosis and a dermal interstitial infiltrate of lymphocytes, and histiocytes. (B) Higher magnification of the dermal interstitial infiltrate with lymphocytes, histiocytes with admixed polymorphonuclear leukocytes including numerous neutrophils and eosinophils.

Histopathological examination of an erythematosquamous plaque confirmed the diagnosis of psoriasis (Figure 4A); skin biopsy from one of the ulcers showed a neutrophil-rich inflammation compatible with PG (Figure 4B). The PARACELSUS score, a currently validated PG diagnostic score, was 19 and confirmed the diagnosis.^1^

In the synopsis of all results, we diagnosed a multilocular PG that was triggered by the acutely exacerbated psoriasis. Therefore, we initiated a systemic immunosuppressive therapy with 1 mg prednisolone/kg bodyweight. Topical therapy of the ulcerations was performed with a polihexanide hydrogel; psoriasis was treated with topical steroids. A considerable improvement was achieved within a few days with the described therapeutic concept. The pain measured by the Visual Analogue Scale decreased from 6/10 to 2-3/10 after 3 days. As the patient never reported any symptoms consistent with an inflammatory bowel disease, we did not perform such investigations during the inpatient stay.

PG is a rare inflammatory neutrophilic dermatosis manifesting most commonly on the lower legs as painful ulcers with violaceous, undermined borders. It is well known that mechanical trauma can lead to the onset of PG. This mechanism is called the pathergy phenomenon.^2^ It is therefore very likely that the PG can be triggered by the inflammatory process of the psoriasis, especially when it worsens, or by scratching of the patient. Furthermore, PG may be associated with malignancies,^3^ which was also the case in our patient.

Up to now, psoriasis has only very rarely been described as a trigger for PG.^4,5^ This rare coincidence is surprising considering that the pathophysiology of PG and psoriasis share many similar features: patients with psoriasis as well as those with PG frequently suffer from comorbidities such as chronic inflammatory bowel diseases or rheumatoid arthritis. These similarities are thought to be based on a common genetic background.^3,6^

In this context, both psoriasis and PG have been discussed as so-called TNF (tumor necrosis factor)-α–associated chronic inflammatory diseases (TRECID).^7^ In these different diseases similar immunological variances in T-lymphocytes have been found,^8^ leading to affection in different body regions. As a clinical consequence, the response of these at first sight markedly different diseases to the same immunomodulating therapies has been described. Both PG and psoriasis respond to systemic therapy with corticosteroids, cyclosporine, or TNF-α inhibitors. Several case reports also showed therapeutic success of treating PG with drugs that specifically block cytokines and are approved for the treatment of psoriasis. These include drugs targeting interleukin (IL)-12/IL-23, IL-17A, and IL-23.^2^ New pathophysiological findings on PG have repeatedly described the association with other autoimmune inflammatory syndromes.^9^ Psoriasis, on the other hand, is considered to be a chronic immune-mediated inflammatory disease. Thus, despite many similarities, there are also considerable pathophysiological differences between the 2 disease patterns.

Acutely exacerbated psoriasis can directly or indirectly trigger PG. Even if this process has been described in case reports very rarely so far, dermatologists and wound care specialists should be aware of this interrelation. Otherwise, the diagnosis might be delayed and the ulcerations may already have progressed. An early joint therapy of both diseases is possible and reasonable.
